# Metabolic risk factors associated with sudden cardiac death (SCD) during acute myocardial ischemia

**DOI:** 10.1080/20961790.2017.1343269

**Published:** 2017-07-06

**Authors:** Dian Wang, Xingxing Wang, Jiayan Wu, Ruibing Su, Jing Kong, Xiaojun Yu

**Affiliations:** Department of Forensic Medicine, Shantou University Medical College, Shantou, China

**Keywords:** Forensic science, forensic pathology, sudden cardiac death, acute myocardial ischemia, tissue metabolomics, metabolic risk

## Abstract

Sudden cardiac death (SCD) is the leading cause of death worldwide. Myocardial ischemia (MI) is the most common underlying causal disorder for SCD. Metabolic risks leading to SCD during acute MI are still not fully understood. Here, using tissue metabolomics, we aimed to investigate myocardial metabolic alterations relevant to SCD events in an acute MI rat model induced by coronary artery ligation (CAL). Thirty-four rats were successfully performed CAL, of which 13 developed lethal ventricular tachyarrhythmia (LVTA)-SCD and 7 developed severe atrioventricular block (AB)-SCD. Fourteen rats that survived within 70 min after the ligation were served as peer controls. The partial least squares-discriminant analysis plots demonstrated clear separations between the SCD rats and controls, indicating obvious differences in myocardial metabolome between these rats. The levels of isoleucine, lactate, glutamate choline, phosphorylcholine, taurine and asparagine in ischemic myocardia were positively associated with LVTA-SCD events; in contrast, the levels of alanine, urea, phenylalanine, linoleic acid, elaidic acid and stearic acid were inversely correlated with LVTA-SCD events. The levels of glutamate and urea were positively and negatively relevant to AB-SCD events, respectively. The dangerous metabolites indicated that lower levels of energy substrates, severe hypoxia, the inhibition of transamination and hyper sympathetic excitement and reactive oxygen species in myocardia were vulnerable to SCD during acute MI. The results suggest fatal metabolic alterations correlated with SCD events during acute MI, which could offer novel clues for the prevention or treatment of acute MI-related SCD.

## Introduction

Sudden cardiac death (SCD) is the leading cause of death worldwide. It accounts for approximately 560 000 deaths and 200 000–450 000 deaths annually in China and the USA, respectively [1,2]. The most common underlying pathological basis for SCD is myocardial ischemia (MI) [3]. Myocardial metabolism would firstly be affected post MI, which may then lead to lethal consequences, including SCD [3]. Several metabolic changes, such as lower ATP and increased level of reactive oxygen species (ROS), have been linked to SCD [4,5]. Metabolic bases inducing SCD under condition of acute MI need deeper exploration. We hypothesized that there may be fatal metabolic changes inducing SCD during acute MI. Therefore, in this study, we developed two SCD rat models elicited by coronary artery ligation (CAL). Using gas chromatography-mass spectrometry (GC-MS) and proton nuclear magnetic resonance (^1^H-NMR)-based tissue metabolomics, we aimed to assess the global difference in myocardial metabolism between the rats who occurred SCD and those who survived during acute MI and analyse myocardial metabolic alterations correlated with SCD events.

## Materials and methods

### Animals and SCD models

This study was approved by the Medical Animal Care & Welfare Committee at our college (Number of authorization: SUMC 2014-157). All procedures were carried out in accordance with the Helsinki Declaration. Adult male Sprague-Dawley rats (weighing 250–400 g) were supplied by the Animal Research Center of our college, and were conventionally fed.

Rats were anesthetized by peritoneal injection of 3% pentobarbital sodium (90 mg/kg, Sigma). After the anesthetized rats were fastened in a rat fixator, they were monitored the lead II electrocardiogram (ECG) using a BL-420 Biological Functional Experimental System (Chengdu Taimeng Co. Ltd., China), then CAL was performed as previously described [6]. In detail, the rats were intubated and ventilated artificially with a small animal ventilator (Shanghai ALCOTT BIOTECH CO., Ltd, China) at 80 strokes/min to maintain normal arterial levels of PO_2_. The inspiratory/expiratory ratio was 1:1 and the tidal volume was 6–10 mL. A thoracotomy was performed horizontally in the fourth intercostal space. The pericardium was opened and the heart was exteriorized. The left coronary artery was ligated 2.0 mm from its origin using surgical suture. The heart was immediately placed back into the thoracic cavity. The ST segment of ECG should rise dramatically after suture, verifying the success of ligation and the occurrence of MI. After MI, some rats developed ventricular tachycardia (VT) and subsequent ventricular fibrillation (VF), and that died thereafter, were designated as the lethal ventricular tachyarrhythmia (LVTA)-SCD group. Some rats did not develop LVTA, but rather developed bradyarrhythmia and then died of severe atrioventricular block, were designated as the atrioventricular black (AB)-SCD group. Other rats that just had paroxysmal arrhythmia, and then returned to a normal sinus heart rate and survived within 70 minutes after suture (the endpoint of the investigation), were served as the peer controls of the SCD groups, and they were euthanized by clamping the aorta. After death, rats’ hearts were immediately retrieved, washed with pre-cooled saline, and stored at −80 °C.

### Metabolic profiling by GC-MS

Myocardial samples were extracted by a previously published procedure [7]. Briefly, 30 mg of myocardium was extracted with 900 μL of methanol/chloroform (*V*_methanol_/*V*_chloroform_, 3:1). After storage at −20 °C for 10 min, the extract was centrifuged at 12 000 g for 10 min at 4 °C (the same below). Two hundred microliter of supernatant was transferred to a GC vial and spiked with an internal standard of D-norleucine (20 µL, 0.5 µmol/mL), mixed well and dried under pure nitrogen blowing in a mild flow velocity at 70 °C. This dried aliquot was subjected to oximation and derivatization procedures with 30 µL methoxiamine (15 mg/mL in pyridine) for 16 h at 20 °C, followed by 30 µL of MTBSFA (1%TMCS) for 1 h at 20 °C. One microliter aliquot of the derivatized solution was separated for GC-MS analysis.

Metabolic profiles were analysed by a GC-MS system consisting of an Agilent 7890N GC system, connected to an Agilent 5975c single quadrupole MSD. A DB-5MS capillary column (Agilent) was used for the separation of metabolites. High-purity helium was used as the carrier gas at a constant flow rate of 1 mL/min. The GC oven temperature was programmed with an initial temperature at 60 °C for 2 min, and then increased lastly to 285 °C at 5 °C /min and maintained for 2 min. The temperature of the injection port, the transfer interface and the EI source were set to 230, 290 and 230 °C, respectively. The selected mass range was set to 50−600 m/z with electron impact ionization (70 eV), and the selected scan speed was 0.99 scans per second.

The GC-MS raw data were processed by XCMS in R language. First, a CDF format file was converted from the chromatogram in each test. After peak matching, retention time correction and peak filling, a txt format file was established for multivariate statistics. Metabolites were identified in the 2.0 NIST library. Peak area of the chromatogram in every sample was normalized using the internal standard peak area.

### Metabolic profiling by ^1^H-NMR

^1^H-NMR-based metabolic profiles were analysed according to a previous study [8]. Briefly, 30 mg of myocardium was disrupted in 0.6 mol/L ice-cold perchloric acid (30 mL/g) by an automated homogenizer and centrifuged, 800 µL of supernatant was collected and neutralized with potassium hydroxide (pH = 7.0), and incubated on ice for 20 min. The supernatant was then frozen at −80 °C and lyophilized for 16 h. The dry powder was dissolved in 550 µL of D_2_O with sodium phosphate buffer (pH = 7.0), vortexed and centrifuged again. Finally, 500 µL of supernatant was transferred to a 5 mm NMR tube for ^1^H-NMR analysis.

^1^H-NMR spectra were obtained at 600.13 MHz (Bruker Biospin, Germany) at 298 K using a NOESYPRID spectrum with water suppression. NOESYPRID spectrum was obtained using the pulse sequence [D1-90°-t-90°-t-90°-ACQ] with a relaxation delay of 4.0 s, a spectral width of 12 335.5 Hz, and 32 scans collected into 16 k data points.

As with standard 1D spectrum, all data-sets were zero filled to 16 k data points and multiplied by an exponential window function with a line broadening apodization factor of 0.3 Hz prior to Fourier transformation. The spectra were manually phased and baseline corrected and chemical shifts were referenced to TSP (0.0 × 10^−6^) using the MestReNova software (version 6.1.0, Mnova). The residual water resonance signal (δ 4.6–5.2) was excluded before analysis. The spectra were “binned” into 0.01 × 10^−6^ regions over the range of 0.5 × 10^−6^−8.2 × 10^−6^, and then the data matrices covering overall metabolic information were produced in an Excel format, followed by a multivariate analysis.

The relative contents of metabolites were calculated with the following formula according to a reference [9]: *C*_m_ = *T*_Im_/*T*_Itsp_ × *N*_p_/9 × *C*_tsp_, where *T*_Im_ and *T*_Itsp_ stand for the total integrals of metabolite and TSP, respectively. *N*_p_ and 9 represent for the number of protons of the metabolite and TSP, respectively. *C*_m_ and *C*_tsp_ stand for the concentrations of metabolites and TSP, respectively.

### Data analysis

The data matrices from ^1^H-NMR and GC-MS were imported into SIMCA-P program (version13.0, Umetrics) for multivariate analysis. The partial least squares discriminate analysis (PLS-DA) was applied with unit variance (UV) scaling. Parameters of the models, such as the *R*^2^*Y* and *Q^2^*, were analysed to ensure the quality of the multivariate models and to avoid the risk of over-fitting.

The associations of myocardial metabolic alterations with SCD events during acute MI were analysed by binary logistic regression using SPSS17.0 software, where the risk ratio (RR) and the 95% confidence interval were calculated.

## Results

### SCD rat model

**Figure 1.**
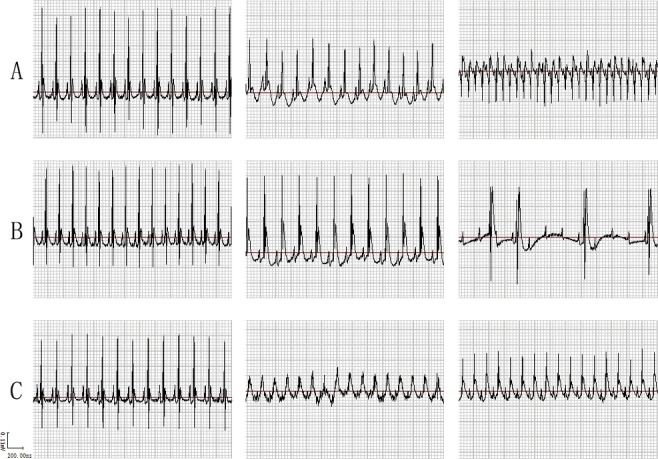
Lead II ECG. (A) LVTA-SCD rats: left and middle, the normal ECG before coronary artery ligation (CAL) operation and ST segment elevation after suture, respectively (the same in the B and C rows); right, ventricular tachycardia and ventricular fibrillation after CAL operation. (B) AB-SCD rats: right, severe atrioventricular block after suture. (C) The controls: right, relatively normal ECG after suture.

Totally, 34 SCD rats were successfully performed CAL to induce acute MI; all had a remarkable elevation of T wave in ECG (Figure 1), confirming the success of CAL and the occurrence of acute MI. Of these, 13 rats developed typical VT and VF, and died from VF (the LVTA-SCD group) from 2.2 to 9.1 min post MI; 7 rats developed bradyarrhythmia post suture, and died from severe atrio-ventricular block (the AB-SCD group) from 2.2 to 4.5 min post MI; 14 rats just had paroxysmal arrhythmia, followed by a normal sinus rhythm within 70 min after suture (controls).

### The difference of metabolic profile between SCD rats and their controls

**Figure 2.**
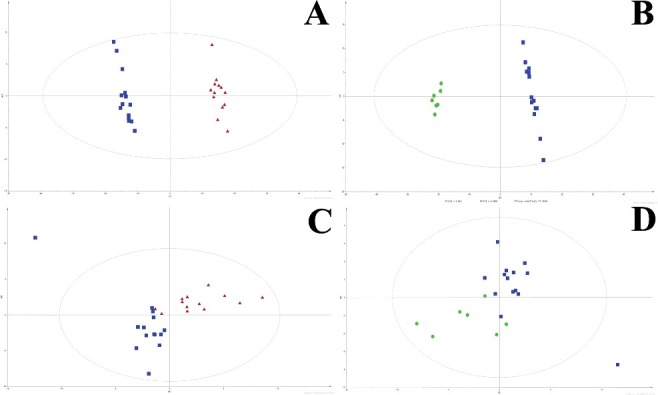
PLS-DA plots. Two-component plots of LVTA-SCD rats (red triangle) and controls rats (blue square) from GC-MS (A) and ^1^H-NMR (C). Two-component plots of AB-SCD rats (green cycle) and controls (blue square) from GC-MS (B) and ^1^H-NMR (D).

The global metabolic differences between the SCD subjects and survival controls were explored by a metabolomics strategy. The PLS-DA models were established by the SIMCA-P 13.0 software using the data both from GC-MS and ^1^H-NMR. Figure 2 shows clear separations between the LVTA-SCD and control groups, and between the AB-SCD and control groups both in GC-MS and ^1^H-NMR modes. The cumulative *R*^2^*Y* and *Q^2^* between the LVTA-SCD and control groups were 0.479 and 0.525, and 0.626 and 0.981 in GC-MS and ^1^H-NMR modes, respectively; those between the AB-SCD and control groups were 0.379 and 0.283, and 0.691 and 0.994 in GC-MS and ^1^H-NMR modes, respectively, when two components were calculated. These results indicate an obvious and significant global metabolic difference between the SCD rats and survival controls during acute MI.

### Risk metabolite change associated with SCD induced by acute MI

To evaluate fatal myocardial metabolic alterations relevant to SCD events during acute MI, we assessed the association between SCD events and the levels of the metabolites identified both in the GC-MS and ^1^H-NMR models. Totally, 42 and 31 metabolites were successfully identified in the GC-MS and ^1^H-NMR modes, respectively. We found that higher levels of isoleucine, lactate, glutamate, choline, phosphorylcholine, taurine and asparagine, and lowered levels of alanine, urea, phenylalanine, linoleic acid, elaidic acid and stearic acid were associated with LVTA-SCD events. Higher level of glutamate and lowered level of urea were related to AB-SCD events. To explore the metabolic changes that were involved in two types of SCDs, we further compared the difference in myocardial metabolites between all SCD rats and the controls, and found that most of the above metabolites were still correlated with SCD events, except for three fatty acids (linoleic acid, elaidic acid and stearic acid) and taurine (Table 1). One possible reason was that these three metabolites had different change trends between the LVTA-SCD and AB-SCD groups, which might offset the significance of the changes when two groups were combined.

**Table 1.** Myocardial metabolites that are associated with the occurrence of SCD post myocardial ischemia.

	^1^H-NMR					GC-MS				
Contrast with controls	Metabolites	FC	*P*	OR	95% CI	Metabolites	FC	*P*	OR	95% CI
AB-SCD	Glutamate	1.27	0.045	30.6	1.08–871.7	Urea	0.69	0.031	0.03	<0.01–0.58
LVTA-SCD	lactate	1.30	0.006	21.6	2.1–218.6	Lactate	1.31	0.046	1.96	1.2–118.9
	Glutamate	1.41	<0.001	21.6	2.1–218.6	Alanine	0.77	0.024	0.05	<0.01–0.51
	Choline	1.33	0.013	5.6	1.1–29.4	Urea	0.67	0.007	0.002	<0.01–0.19
	Pho-choline	1.28	0.013	20.1	2.8–145.3	Glutamate	1.52	0.032	17.4	1.28–239.2
	Taurine	1.17	0.049	13.8	2.1–92.0	Phenylalanine	0.76	0.042	0.47	0.23–0.97
	Isoleucine	1.29	0.009	12.0	1.2–118.9	Asparagine	1.56	0.027	1.41	1.04–1.92
						Linoleic acid	0.56	0.025	0.29	0.098–0.86
						Elaidic acid	0.58	0.038	0.083	0.008–0.87
						Stearic acid	0.72	0.035	0.46	0.23–0.95
SCD	isoleucine	1.30	0.004	9.0	1.5–52.3	Lactate	1.31	0.046	1.96	1.2–118.9
	lactate	1.24	0.010	12.0	1.9–72.9	Alanine	0.79	0.009	0.018	0.01–0.61
	Glutamate	1.36	<0.001	7.2	1.5–33.8	Urea	0.68	<0.001	0.02	<0.01–0.35
	Choline	1.32	0.008	9.0	1.6–51.5	Phenylalanine	0.79	0.046	0.47	0.24–0.94
	Pho-choline	1.25	0.008	11	2.2–56.1	Glutamate	1.19	0.034	9.82	1.18–81.4
						Asparagine	1.42	0.047	1.27	1.01–1.62

Note: FC, fold change, the ratio of myocardial metabolite's relative content in SCD rats to that of control rats; *P*, significance of association analysis; OR, odds ratio calculated by binary logistic regression in SPSS; 95% CI, 95% confidence interval for OR.

## Discussion

In this study, we compared myocardial metabolic difference between the rats who died from SCD and those who survived during acute MI. First, using the tissue metabolomics, we investigated the global metabolic difference between them. The PLS-DA scores demonstrated clear separations between the LVTA-SCD rats and controls, and between the AB-SCD rats and controls, both from GC-MS and ^1^H-NMR mode. The results suggest significant differences in myocardial metabolic profile between the SCD rats and the controls.

Totally, 13 metabolites were associated with LVTA-SCD events during acute MI. Of these, linoleic acid, elaidic acid and stearic acid were down-regulated in myocardia of the LVTA-SCD rats. Because fatty acids are the major energy substrates of the heart [10,11], the lower levels of these fatty acids indicated myocardial energy deficit of these rats. Under condition of acute MI, the depletion of energy in myocardia may accelerate the process of death; in which the occurrence of ventricular arrhythmia and its successive heart failure may be the most important mechanism. Accumulating evidences suggest cardiac ion channel/transporter dysfunction is closely linked to abnormal myocardial energy metabolic activity [3]. An explanation is that lower energy in myocardia cannot maintain cardiac ion balance owing to a lower activity of ion-related ATPase. Then, the resulting cardiac ion imbalance may lead to lethal arrhythmia. Additionally, we found higher choline and phosphorylcholine levels in myocardia of the LVTA-SCD rats, which suggested that myocardial membranes of these subjects were destroyed. The dysfunction of myocardial membranes could alter their fluidity, then eliciting lethal arrhythmia [12,13]. Lactate is a product of glycolysis. It has been proved to be remarkably increased during acute MI [14]. We found higher lactate level in myocardia of LVTA-SCD rats compared to the survival controls, implying that these SCD rats experienced even more severe hypoxia and energy depletion. Isoleucine is a branched-chain amino acid (BCAA). BCAA catabolism was inhibited under condition of heart failure [15], which could explain the high isoleucine level in myocardia of LVTA-SCD rats because the SCD rats also experienced a process of heart failure, which then might impair bioenergetic regulation in the heart, accelerating LVTA-related the process of death.

Glutamate and taurine were increased, while urea and alanine were declined in myocardia of the LVTA-SCD rats compared to the survival controls. A previous study showed that the uptake of glutamate in myocardia was enhanced under condition of MI, which would serve as a NH_2_ donor [16]. The current results suggested that the transamination may be relatively prohibited, namely, less NH_2_ is transferred from glutamate to urea or alanine in myocardia of LVTA-SCD rats. It also indicated that if more NH_2_ existed as a toxic form, such as the higher level of glutamate in myocardia, the subject would be vulnerable to LVTA-SCD during acute MI. Circulating glutamate and taurine was recently found to be associated with the generation of ROS in paroxysmal atrial fibrillation [17], indicating that glutamate and taurine is able to induce arrhythmia through the production of ROS, inducing LVTA-SCD. In addition, higher glutamate level was proved to be correlated with poor prognosis in cerebral acute ischemic stroke [18], and the latter has a similar pathological condition with acute MI, which suggested that higher glutamate level was a detrimental metabolic change in ischemic conditions and it should play a key role in the occurrence of lethal arrhythmia and SCD during acute MI. Phenylalanine is a substrate of catecholamines; its decline in myocardia of LVTA-SCD rats suggests more norepinephrine and epinephrine are produced and the excitement of sympathetic nerves during the process of LVTA induced by acute MI [19].

However, only glutamate and urea were found to be associated with AB-SCD, both of which were also relevant to LVTA-SCD. The result of urea was contrary to that of one clinical research, which found that serum urea increased the mortality of heart failure patients [20]. It can be explained as that urea is related to overall clinical status of the heart failure patients, including renal, hemodynamic and neurohormonal parameters. However, a lower level of urea was more indicative of less transamination in ischemic myocardia, where glutamate was higher and more NH_2_ exist as a toxic form. Compared to controls, these AB-SCD rats might have no extra energy depletion, hypoxia, sympathetic excitement and ROS production because the principal mechanism for AB-SCD during acute MI was heart failure, similar to controls in this study [21].

## Conclusion

There were obvious differences in myocardial metabolome between the LVTA-SCD, AB-SCD rats and their peer controls during acute MI. Under acute MI, lethal myocardial metabolic mechanisms that associated with LVTA-SCD may be lower levels of energy substrates, severe hypoxia, sympathetic excitement, ROS generation and inhibition of transamination. The latter was also found to be relevant to AB-SCD. The fatal metallic changes identified here may offer novel clues to discover potential biomarkers for SCD-related forensic practices and to find therapeutic targets for SCD induced by acute MI.
